# Why not both? A case study in measuring cortisol and corticosterone

**DOI:** 10.1093/iob/obaf041

**Published:** 2025-11-19

**Authors:** S E Westrick, R T Paitz, E K Fischer

**Affiliations:** Department of Biology, Wake Forest University, Winston-Salem, NC 27103, USA; Department of Evolution, Ecology and Behavior, University of Illinois Urbana-Champaign, Urbana, IL 61801, USA; School of Biological Sciences, Illinois State University, Normal, IL 61790, USA; Department of Evolution, Ecology and Behavior, University of Illinois Urbana-Champaign, Urbana, IL 61801, USA; Department of Neurobiology, Physiology, and Behavior, University of California Davis, Davis, CA 95616, USA

## Abstract

A general tenet in stress physiology is that the hypothalamic-pituitary-adrenal axis predominantly produces one glucocorticoid (GC) in response to stressors. However, most vertebrates produce both cortisol and corticosterone, these steroids show variation across species in absolute levels, relative proportions, and stress responsivity, and they regulate much more than just stress physiology. In the present commentary, we argue that focusing on a single GC may not capture the whole story, presenting an overview of previous studies and an example from our own work on poisons frogs—a group relatively new to endocrinological studies. We originally set out to validate non-invasive waterborne hormone measurements in our focal species, the dyeing poison frog *Dendrobates tinctorius*. In pursuing this goal, we uncovered unexpected patterns of GC abundance within and across species. *Dendrobates tinctorius* had higher amounts of corticosterone than cortisol in both plasma and waterborne samples, and corticosterone was responsive to adrenocorticotropic hormone as canonically assumed. However, corticosterone and cortisol levels were surprisingly similar in *D. tinctorius*, and cortisol was more abundant than corticosterone in water samples from four additional poison frog species. Alongside those of other studies, these results challenge the broadly accepted assumption that corticosterone is always more abundant in amphibians and add to the growing literature highlighting the importance of measuring both GCs to understand (stress) physiology.

## Introduction

The ability to measure physiology non-invasively in the lab and in the wild is key for behavioral ecologists and conservation biologists seeking to understand animal behavior in a changing world. Glucocorticoids—most commonly cortisol and corticosterone produced by the adrenal or interrenal glands (Sapolsky et al. 2000)—are of keen interest because they elicit a variety of physiological, morphological, and behavioral responses (MacDougall-Shackleton et al. 2019). Glucocorticoids are often measured from plasma samples but can also be measured in feces, urine, hair, feathers, baleen, eggs, and water (Narayan et al. 2019; Palme 2019). These non-invasive sampling methods can reduce the confounds of stress from sampling itself and allow for repeated measures from small individuals where multiple blood draws are not possible or when individuals cannot be captured (Palme 2019).

The majority of studies across taxa measure only a single GC, the one considered to be “dominant,” as a readout of stress (Hancock 2010). Which GC is dominant varies across species (Koren et al. 2012), with the general assumption that this variation is not functionally interesting because the two GCs are physiologically redundant. Yet, other glucocorticoids are present, potentially in substantial amounts, and can be produced independently of the hypothalamic-pituitary-adrenal (HPA)/I axis (Bornstein et al. 2008), for example, locally in different tissue types (Schmidt and Soma 2008). Moreover, there is intriguing evidence that GCs long assumed to be physiologically interchangeable may in fact play distinct, specialized roles (e.g., Schmidt and Soma 2008). To fully understand the complex roles glucocorticoids play, we need to move beyond studying a single GC to characterization of multiple GCs that together regulate behavior, development, and individual and population health.

In the present article, we review evidence for the importance of measuring both glucocorticoids—especially in species new to endocrinological study—using our own work as an example. We set out to validate non-invasive waterborne hormone measurements in our focal species, the dyeing poison frog *Dendrobates tinctorius*. In pursuing this goal, we uncovered unexpected patterns of cortisol and corticosterone abundance within and across species of poison frogs, with implications for how we think about and quantify GC physiology. Alongside a growing literature, we briefly review, our findings highlight the value of measuring both cortisol and corticosterone, specifically in frogs and for those interested in behavior and conservation across vertebrates more broadly.

### Cortisol vs. corticosterone

Cortisol and corticosterone are best known for their role in the “stress response” during which they coordinate behavioral, physiological, and metabolic responses. However, they also change with circannual and circadian rhythms (Dickmeis et al. 2013) and play a critical role in other physiological processes (MacDougall-Shackleton et al. 2019), including, but not limited to, energy metabolism (Vegiopoulos and Herzig 2007), immune response (Cruz-Topete and Cidlowski 2015), growth and development (Seckl and Meaney 2004), reproduction (Whirledge and Cidlowski 2017), and cardiovascular function (Cruz-Topete et al. 2016). Circulating GCs also impact behaviors, including aggression (Haller 2014), parental care (Bales et al. 2006; Crossin et al. 2012; Raulo and Dantzer 2018), and feeding/foraging (Crespi and Denver 2005; Crossin et al. 2012). Because of their widespread influences, changes in GC levels and/or their receptors can be immensely impactful on survival and fitness. While acutely elevated GC levels often have adaptive functions, chronically elevated GCs disrupt normal physiology and decrease fitness (reviewed in Bonier et al. 2009 and Breuner et al. 2008).

**Fig. 1:**
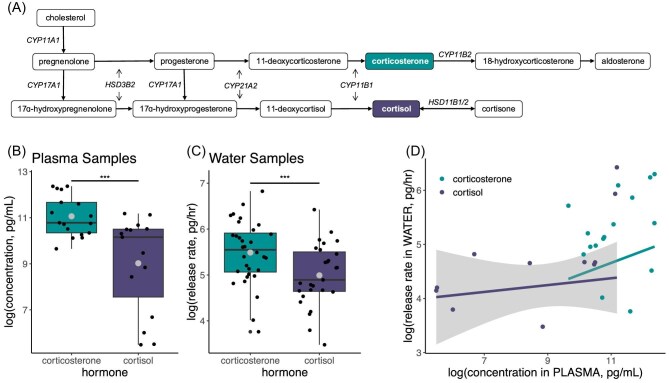
(A) Cortisol and corticosterone are produced through similar pathways which share biosynthetic enzymes. However, the pathway to produce cortisol requires the hydroxylase enzyme cytochrome P450 17A1, encoded by the gene *CYP17A1*, to convert pregnenolone to 17ɑ-hydroxypregnenolone or convert progesterone to 17ɑ-hydroxyprogesterone. Notably, corticosterone is not converted directly into cortisol or vice versa. Corticosterone was more abundant in both (B) plasma and (C) water samples from *D. tinctorius*. Larger dots indicate the mean for each treatment group. Smaller dots are individual water samples. (*** = *P* < 0.001). (D) The concentration of cortisol, but not corticosterone, in plasma was positively correlated with the concentration in water samples from the same individuals. Gray shaded area is the confidence interval around the statistically significant linear regression for cortisol.

Many vertebrates produce both cortisol and corticosterone. As steroid hormones, cortisol and corticosterone are produced from cholesterol (Fig. 1A) (Payne and Hales 2004). While these pathways share precursors, the final synthesis steps are non-overlapping and the two GCs cannot be directly converted from one to the other (Fig. 1A). Typically, either cortisol or corticosterone is considered the dominant GC, defined as the GC released in response to adrenocorticotropin hormone (ACTH). Therefore, ACTH challenges are commonly used to measure the responsiveness of the adrenals, to identify the dominant GC, and to validate that a chosen method of hormone measurement is detecting an increase in HPA axis activity (Touma and Palme 2005; Palme 2019). When ACTH challenges are not possible, dominance is often assumed based on higher abundance (Aerts 2018), resulting in conflation of dominance and abundance. Because both cortisol and corticosterone bind to glucocorticoid and mineralocorticoid receptors, they are generally assumed to be interchangeable in their physiological function (e.g., Funder 1993).

Due to taxonomic patterns and broadly accepted assumptions of physiological interchangeability, we often make assumptions about which GC is dominant, and therefore assumed to be more abundant, in our species of interest. Consequently, many studies measure only one GC, such as cortisol in fish or corticosterone in birds. These simplifying assumptions are especially prevalent in studies using GCs as a readout of stress or correlate of behavior, rather than exploring GC physiology per se. Yet, these assumptions and generalizations can be misleading.

GC dominance and the ratio of corticosterone to cortisol varies both across (Koren et al. 2012) and within (e.g., Larson et al. 2009; Koren et al. 2012; Keogh et al. 2020) species, and a growing number of studies demonstrate the importance of testing interspecific variation in GCs even among closely related species. For instance, across mammals, we see all combinations of which GC responds to an acute stressor or exogenous ACTH, independent of relative abundance. Some mammals respond primarily with an increase in cortisol (Ganjam et al. 1972; Van Mourik et al. 1985; Vera et al. 2011, 2012; Johnston et al. 2013), while in others corticosterone increases (Young et al. 2004), and still in others *both* GCs respond (Smith and Bubenik 1990; Rosenthal et al. 1993; Young et al. 2004). Sea otters (*Enhydra lutris*) are an interesting case where some studies document an increase in cortisol in response to ACTH (Wasser et al. 2000; Murray et al. 2020), while others find an acute stressor (catching and handling) causes an increase in corticosterone (Larson et al. 2009). These findings suggest a potential for non-ACTH regulation of corticosterone, such as modulation by epinephrine or norepinephrine (Bornstein and Chrousos 1999). This within and among species variance in abundance and responsiveness suggests that we should use caution when following traditional assumptions about which GC to measure based on order, family, or even population within the same species (Hancock 2010).

More importantly, the GC that is less abundant or not responsive to ACTH can still play an important physiological role. For instance, in humans, cortisol is considered the primary effector hormone of the HPA axis (Brück 1983); however, corticosterone plays a role in the central nervous system (Raubenheimer et al. 2006) and pre-natal responses to stressors (Wynne-Edwards  et al. 2013). Corticosterone is also an intermediary in the production of aldosterone and the rate of conversion from corticosterone to aldosterone may vary resulting in more or less circulating corticosterone in cortisol-dominant species (Koren et al. 2012). Additionally, steroid receptors and transporters can interact with cortisol and corticosterone differently. In fact, the two GCs can elicit different effects due to different receptor binding affinities (Sutanto and De Kloet 1987; Karssen et al. 2001). By measuring only one GC, we miss out on finding species that produce measurable amounts of both, miss opportunities to test hypotheses about the independent regulation and function of cortisol vs. corticosterone, and do not have a complete picture of physiological regulation in health and disease.

### Glucocorticoids in amphibians

Environmental and anthropogenic changes are contributing to amphibian population decline and rapid biodiversity loss (Kiesecker et al. 2001; Blaustein and Kiesecker 2002; Stuart et al. 2004; Collins 2010; Kiesecker 2011; Ohmer et al. 2021). Many amphibians are small bodied and developing non-invasive techniques to study their stress physiology is instrumental in understanding how species respond to multifactor environmental stressors, such as habitat modification, climate variability, pollution, and disease, and can inform conservation strategies for at-risk species (Narayan 2013; Kumar and Umapathy 2019; Palme 2019; Walls and Gabor 2019). GCs also play critical roles in metamorphosis in amphibians (Denver 2009; Kulkarni and Buchholz 2014) with size at metamorphosis having life-long consequences for survival and fitness (Glennemeier and Denver 2002; Kulkarni and Gramapurohit 2017).

Corticosterone is typically referenced as the dominant and vastly more abundant glucocorticoid in amphibians (Sandor 1969; Moore and Jessop 2003; Wingfield and Romero 2011; Norris and Carr 2013), and occasionally listed as amphibians’ only glucocorticoid (Katsu and Iguchi 2016). In the 1970s and 1980s, early studies of steroid synthesis reported production of corticosterone and little, if any, cortisol from isolated interrenal cells of frogs and toads *in vitro* (Chan and Edwards 1970; Mehdi and Carballeira 1971; Jolivet-Jaudet and Ishii 1983; Jolivet-Jaudet and Leloup-Hatey 1984). The studies from just a few species of frogs and toads (namely *Bufo bufo. Rana catesbeiana*, and *Xenopus laevis*) led to a strong focus on corticosterone in most studies on stress physiology across all amphibians.

This bias toward studying corticosterone in amphibians prevails despite a history of cortisol being detected in several species of anurans (frogs and toads) (e.g., Dale 1962; Krug et al. 1983) and a growing number of studies demonstrating that the assumption of corticosterone responsiveness to ACTH/acute stressors and/or greater corticosterone abundance does not hold for all amphibians (e.g., Baugh et al. 2018). For instance, in fecal samples from two species of Panamanian harlequin frogs (*Atelopus certus* and *A. glyphus*), both cortisol and corticosterone were responsive to ACTH, but cortisol metabolites were detected at much higher concentrations overall (Cikanek et al. 2014). In the common water frog (*Rana esculenta*), cortisol and corticosterone are both detectable in plasma and both are elevated in ovulatory and postovulatory phases compared to the preovulatory phase (Gobbetti and Zerani 1993).

In an attempt to resolve these discrepancies, some researchers have suggested that aquatic amphibia may rely on cortisol as their primary GC, as many fishes do, whereas semi-terrestrial or terrestrial amphibia use corticosterone (Jungreis et al. 1970). For example, aquatic hellbender salamanders (*Cryptobranchus alleganiensis*) mainly produce cortisol in response to a stressor (Hopkins et al. 2020). Yet, the vast majority of studies on a popular aquatic model species, the clawed frog (*Xenopus* sp.), measure corticosterone (e.g., Jaudet and Hatey 1984; Kulkarni and Buchholz 2012; Shewade et al. 2020), likely based on foundational *in vitro* studies with *X. laevis* interrenal cells (Chan and Edwards 1970; Jolivet-Jaudet and Leloup-Hatey 1984). Conversely, in samples from dermal swabs, cortisol increases in response to ACTH in terrestrial green treefrogs (*Hyla cinerea*), American toads (*Anaxyrus americanus*), and red-spotted newts (*Notophthalamus viridescens*) (Santymire et al. 2018). Further equivocal results about cortisol and corticosterone in amphibians, including contradictory results in the same species (such as *X. laevis* and *Rana pipens*), are nicely summarized in Hopkins et al. (2020).

### A case study on stress physiology in Neotropical poison frogs

Neotropical poison frogs (Dendrobatidae) have long garnered interest from evolutionary biologists and hobbyists, and have gained recent attention in behavioral ecology and neuroethology (Pašukonis et al. 2014; Roland and O’Connell 2015; Burmeister 2022; Ringler et al. 2023; Westrick et al. 2023; Peignier et al. 2024). These often brightly colored, primarily diurnal, terrestrial frogs are threatened by agrochemicals (Angulo et al. 2019), climate change (Whitfield et al. 2007), smuggling (Auliya et al. 2016), habitat destruction or fragmentation (Cushman 2006; Becker et al. 2007), and susceptibility to the amphibian chytrid fungus *Batrachochytrium dendrobatidis* (Courtois et al. 2015). Due to their conservation status (Guillory et al. 2019) and generally small body size, with an average snout-vent length (SVL) of ∼17–60 mm, repeated plasma sampling is not feasible, and there is a need to further develop non-invasive methods in the wild and the lab.

Our goal was to characterize GC abundance and ACTH-responsivity in adult dyeing poison frogs, *Dendrobates tinctorius*, which have received growing interest in behavioral and evolutionary studies in recent years (Rojas and Pašukonis 2019; Moskowitz et al. 2022; Pašukonis et al. 2022; Sabino-Pinto et al. 2024; Schlippe Justicia et al. 2024; Soto et al. 2024; Mayer et al. 2025). We previously found corticosterone is more abundant and ACTH-responsive in *D. tinctorius* tadpoles (Surber-Cunningham et al. 2025), but also found a surprising role for cortisol in parental behavior (Fischer and O’Connell 2020).

To build a more complete picture of poison frog physiology, we used non-invasive waterborne hormone sampling to allow for repeated measures from the same individuals and an ACTH challenge to assess the responsivity of the HPA axis to ACTH (e.g., Gabor et al. 2013; Baugh et al. 2018; Rodríguez et al. 2022). We then compared glucocorticoid concentrations between water and plasma samples from the same individuals to assess whether water samples are representative of circulating hormone levels in blood. To test if the patterns we found were consistent across Dendrobatids, we also characterized cortisol and corticosterone excretion in four additional, related poison frog species.

## Materials and methods

### Animal husbandry

We housed all frogs in our breeding colony at University of Illinois Urbana-Champaign in glass terraria (18 × 18 × 18″, 12 × 12 × 18″, or 36 × 18 × 18″ Exo Terra^®^) with soil, sphagnum moss, live tropical plants, and coconut shelters. We kept frogs on a 12:12 h light cycle and fed them flightless *Drosophila* fruit flies (*D. melanogaster* or *D. hydei*) dusted with vitamin supplements three times weekly. Our automated RO water misting system kept tank humidity >75% and the room was kept at 21–24°C. In addition to *D. tinctorius*, we measured GCs in golden poison frogs (*Phyllobates terribilis*), mimic poison frogs (*Ranitomeya imitator*), Zimmermann’s poison frogs (*R. variabilis*), and Anthony’s poison frogs (*Epipedobates anthonyi*). These species share many behaviors and are endemic to tropical regions of Central and South America, but vary in body size and toxicity in the wild (Wells 2007). *Phyllobates terribilis* and *D. tinctorius* are among the largest poison frogs (∼37–60 mm SVL; ∼4–6.4 g), *E. anthonyi* are intermediate (∼19–25 mm SVL; ∼0.75–1.4 g), and both *Ranitomeya* are among the smallest (∼17–22 mm SVL; ∼0.4–0.6 g). We housed adult frogs in groups of two to three individuals of the same species, either one or two males with one female (*E. anthonyi. D. tinctorius. R. variabilis*, and *R. imitator*) or as a group of two to three frogs that were too young to be sexed at the time of sampling (*P. terribilis*). All frogs were at least 1 year old. For dosing during the ACTH challenges in *D. tinctorius*, we weighed frogs prior to sampling by placing them in a plastic container on a scale and subtracting the weight of the container. All procedures were approved by UIUC Institutional Animal Care and Use Committee (protocol #20147).

### Hormone sampling

To collect waterborne hormones, we placed individual frogs in glass containers with room temperature RO water. For the smaller species (*E. anthonyi. R, variabilis*, and *R. imitator*), we used 4 cm × 4 cm × 5 cm hexagonal glass jars with lids filled with 15 mL of RO water. For the larger frogs (*D. tinctorius* and *P. terribilis*), we used 6.5 cm × 6.5 cm × 6 cm glass tubs with lids and 40 mL of RO water. Our goal was to use enough water to cover frogs’ highly vascularized pelvic skin in a container small enough that the frogs would stay in the water and not climb the walls. We left frogs in the water for 1 h before returning them to their home terraria or a new water tub for additional sampling. We collected all samples 4.5–8 h into the frog’s 12 h light cycle. We filtered water samples with Whatman^®^ 1 filter papers to remove any large particulates, prior to steroid hormone extraction (see Section “Steroid hormone extraction”). To compare across species, we collected water samples from *D. tinctorius. E. anthonyi. P. terribilis. R. imitator*, and *R. variabilis*. We stored all water samples at −20°C until extraction. For the plasma and waterborne hormone comparison, we collected water samples as described above for *D. tinctorius* immediately prior to collecting plasma. After thorough vortexing, we subsampled half of the water from *n* = 155 water samples for validations and stored each subsample separately. To collect plasma samples, we anesthetized frogs with benzocaine (Orajel™) and euthanized via rapid decapitation. This process took <5 min, in line with generally accepted time courses for plasma sample collection. We used heparinized microhematocrit capillary tubes to collect trunk blood from the body. We combined all the collected blood from an individual in a heparinized microcentrifuge tube and centrifuged for 5 min at 5000 *g* to separate the plasma. Using a micropipette, we collected the plasma supernatant and stored all plasma samples at −20°C.

### ACTH hormone challenge

For the ACTH hormone challenges, we used a within-individual repeated measures design with 18 adult *D. tinctorius* (9 male, 9 female) for a total of 36 challenges (18 ACTH injections and 18 control injections). We injected frogs with ACTH (Sigma–Aldrich^®^, product no. A7075) diluted in 0.9% saline and a vehicle injection of only 0.9% saline with 6 days between injections. The order of injections was counterbalanced across individuals such that half of the frogs were first injected with vehicle, and half were first injected with ACTH. Prior to the start of trials, we dissolved the ACTH in saline at a concentration of 0.125 ug/µL and aliquoted the solution into insulin syringes which were frozen at −20°C until the day of injection, along with saline vehicle aliquots. On the day of the trial, we thawed the aliquots of saline and ACTH and calculated the volume needed to inject each frog with a dosage of 0.5 μg ACTH per g body weight (mean ± SD = 5.89 ± 1.5 g), based on previous work using ACTH challenges in Neotropical frogs (Baugh et al. 2018). Since the precision of the insulin syringes was limited, we rounded to the nearest 10 µL to get the volume for injection with both ACTH and saline. For each trial, we collected a waterborne hormone sample, as described above, for 1 h prior to injection as our “pre-injection” sample. We then used insulin syringes to inject frogs intraperitoneally. After injecting, we immediately placed the frogs in a new water bath for one hour to collect the “post-injection” sample. After the post-injection sample, we returned frogs to their home terraria. We repeated this process the following week with the alternate treatment (ACTH or saline). We performed all trials between 5 and 7 h after the lights turned on. We vortexed each water sample immediately prior to splitting the water (four samples per 18 frogs = 72 samples total) and stored subsamples separately.

### Steroid hormone extraction

For all water samples, we used C18 cartridges (Waters™ Corporation, Milford, MA, USA) for solid-phase extraction to collect total steroid hormones (Gabor and Grober 2010; Gabor et al. 2013; Baugh et al. 2018; Kim et al. 2018). To test the recovery rate of the cartridges, we spiked clean water samples with a known quantity of hormone and found a 101% and 91% average recovery of cortisol and corticosterone, respectively. With cartridges on a vacuum manifold, we used a vacuum pump to prime the filters with 6 mL 100% methanol. We then rinsed the cartridges with 6 mL RO water before passing the sample through the cartridge. We eluted the hormones from the cartridges with 4 mL of 100% methanol into 13 × 100-mm borosilicate glass vials. We dried samples under a stream of nitrogen gas in a 37°C dry heating block until completely dry. We stored desiccated samples at −20°C until reconstituted with assay buffer for enzyme linked immunosorbent assay (ELISA) measurement of total steroid hormones (see Section “Enzyme linked immunosorbent assays”).

To measure glucocorticoids from plasma samples, we used the dissociation reagent included in our ELISA kits (see Section “Enzyme linked immunosorbent assays”). Due to the limited volume of plasma collected from each frog (∼50–100 µL), we chose to use the dissociation reagent over doing an extraction process due to small sample volumes and to avoid losing sample through the extraction steps. We combined 5 µL of dissociation reagent and 5 µL of plasma, vortexed, and incubated for a minimum of 5 min at room temperature. We then added 450 µL of assay buffer to make a 1:92 dilution before running samples on ELISA plates.

### Enzyme linked immunosorbent assays

We used DetectX^®^ ELISA Kits from Arbor Assays™ (Ann Arbor, MI, USA) to measure total cortisol and corticosterone concentrations in plasma and water samples. We used each kit according to the manufacturer’s instructions, including using the same assay buffer (catalog no. X065) for both kits to allow us to split samples across cortisol and corticosterone plates. For the corticosterone kit, we followed instructions for the 100 µL format because the standard curve for the 100 µL format can measure smaller concentrations than the 50 µL format. To run samples across both plates, we reconstituted samples in 225 µL assay buffer, pipette samples on the cortisol plate, then added 100 µL of assay buffer before pipetting sample on the corticosterone plate. After each addition of assay buffer, the samples were thoroughly vortexed. For both cortisol and corticosterone, we measured the optical density at 450 nm. After removing samples with a coefficient of variation (CV) >20% (*n* = 77 corticosterone and *n* = 62 cortisol, out of 241 measurements each), the average intra-assay CV across the remaining samples for the analyses was 8.2% for cortisol and 8.1% for corticosterone. The cross reactivity for the cortisol ELISA plate with corticosterone is reported by the manufacturer as 1.2% and for the corticosterone ELISA plate cross reactivity with cortisol is reported as 0.38%. Information about cross reactivity with other molecules can be found on the Arbor Assays™ website.

We used a four-parameter logistic curve regression to fit the respective standard curve on each plate using MyAssay software recommended by the plate manufacturer (www.MyAssays.com). Based on this curve, we calculated the concentration of cortisol and corticosterone (pg/mL of assay buffer) for each sample and averaged across duplicates. Due to low concentrations of both GCs in some species, some samples were too low to fall on the standard curve (*n* = 13 corticosterone and *n* = 30 cortisol, out of 241 measurements each), though some had concentrations of cortisol that were too high (*n* = 10). To be conservative, for samples that were off the standard curve in either direction (too low or too high based on the lowest and highest standards), we censored the data at the minimum or maximum detection limits of the assay, respectively. To confirm the censored data did not skew our findings, we ran additional analyses with only samples measuring within the assay detection limits and CV <20%, and the overall pattern of results remained the same. We report release rate (pg/h) for both cortisol and corticosterone from water samples (Gabor et al. 2013). We calculated release rate by multiplying the concentration calculated from the standard curve on the plate by the volume of buffer used to reconstitute the sample. This gave us the approximate total mass (pg) of hormone in the original water sample which we divided by the duration of collection to get release rate (pg/h). For samples that were split in half prior to extraction, we multiplied the rate (pg/h) by two to allow comparison with samples that were not split.

We tested each assay for parallelism by combining 24 additional water samples from adult *D. tinctorius* to create a highly concentrated pooled sample and then serial diluting by 1/2 of the reconstituted extracts for a dilution curve with seven samples in total. This serial dilution sample was split four ways to test parallelism across four different ELISA plates for steroid hormones, including cortisol and corticosterone. We confirmed parallelism by comparing the slope of the serially diluted curve to the slope of the standard curve using an interaction term in a linear model. We found the dilution curves were not significantly different from the standard curve for their respective hormone (cortisol: ß = −0.34, SE = 1.08, *P* = 0.76; corticosterone: ß = −5.09, SE = 3.06, *P* = 0.12).

### Statistical analysis

We used R v4.2.2 (R Core Team 2024) in RStudio v3.1.446 (Posit Team 2025) for all statistical analyses and ran all analyses on log transformed hormone release rates to normalize residuals.

First, we compared the concentration of cortisol and corticosterone in plasma and water from *D. tinctorius*. We calculated the original plasma sample concentration by multiplying the concentration measured in the assay by the dilution factor. For the analyses with water samples alone, we included all unmanipulated samples, including those taken immediately prior to euthanasia and samples taken on the first day of trials before the injection during ACTH challenges. We fit two separate linear models (R packages lme4 (Bates et al. 2015) and lmerTest (Kuznetsova et al. 2017)) with concentration (pg/mL) in plasma (*n* = 32 samples, 17 frogs) and hormone release rate (pg/h) in water samples (*n* = 59 samples, 32 frogs). For both models, we included GC type (cortisol or corticosterone) and sex as fixed effects and frog ID as a random effect to control for repeated samples, since both hormones were measured in each sample.

Next, we were interested in whether the GC types are correlated with one another which would suggest measuring either one is sufficient in most scenarios. To assess the correlation between GC types, we fit two linear models (plasma model, *n* = 15 samples; water model, *n* = 23 samples) with cortisol concentration or release rate as the response variable and corticosterone as the fixed effect. Additionally, to examine the relationship between waterborne glucocorticoids and circulating glucocorticoids in plasma, we fit linear models for both cortisol (*n* = 11 frogs) and corticosterone (*n* = 16 frogs), separately, with plasma concentrations (log transformed pg/mL) as the fixed effect predicting water hormone release rate (log transformed pg/h) for the matched plasma-water samples.

For the ACTH challenges, we fit a linear mixed effects model to ask if there was an effect of treatment (ACTH or control) on either cortisol or corticosterone. The model had the following main effects: SVL in cm, GC type (cortisol or corticosterone), time point (pre- or post-injection), and injection type (ACTH or saline). Because these data were all collected from one species, we included SVL in the model to account for differences in hormone release rate due to body size within species, as is recommended in waterborne hormone sampling in fish (Scott and Ellis 2007). We chose SVL over mass to avoid variation in hydration levels which can have a substantial effect on body mass in amphibians. We also included a three-way interaction between GC type, time point, and injection type. This interaction indicated whether the difference between pre- and post-injection varied by injection type and if this relationship varied by GC type. In other words, was there a change in cortisol from pre- to post-injection based on injection type and was this change (or lack thereof) the same or different in corticosterone? We included trial ID as a nested random effect within frog ID to account for repeated measures across trials and individuals. We used post-hoc pairwise comparisons using the multcomp package (Hothorn et al. 2008) to interpret interaction effects. After filtering for CV, our sample size was 26 pre- and 26 post-injection cortisol measurements and 27 pre- and 30 post-injection corticosterone measurements. Because our main interest was how the within-individual response to the injection varied across injection types, all four samples for each individual were run on the same plate to reduce possible confounding effects of inter-plate variation within individuals and trials.

Across species, we were primarily interested in whether the release rates of the two GCs differed within species, rather than absolute GC differences between species. To this end, we fit a linear mixed model for log transformed hormone release rate with a fixed effect of the interaction between species and glucocorticoid type (corticosterone vs. cortisol) with sample ID as a random effect. We ran a Type III analysis of variance (ANOVA) on the linear mixed model to assess the overall effect of the interaction term. We then ran post-hoc simultaneous pairwise comparisons using the multcomp package (Hothorn et al. 2008) to specifically look at the difference in release rate between cortisol and corticosterone within species (e.g., *P. terribilis* cortisol vs. *P. terribilis* corticosterone). We did not include SVL as a correction for body size, as the large species differences in average body size confound the effect of SVL and species if both are included in the same model. Furthermore, SVL was not significantly correlated with GCs in our *D. tinctorius* specific analyses (Table 1), which is consistent with previous studies on waterborne hormones in frogs (e.g., Gabor et al. 2013). We also did not include sex as a covariate since not all individuals could be confidently sexed at time of sampling. However, in a reduced model with only individuals that we could confidently sex, we found no overall effect of sex on GC production (*F*_1,79.5_ = 0.033, *P* = 0.86). We were primarily interested in comparing the abundance of GCs within species rather than across species. Our final species comparison dataset included 59 measurements from *D. tinctorius* (32 corticosterone, 27 cortisol), 37 measurements from *E. anthonyi* (15 corticosterone, 22 cortisol), 23 measurements from *P. terribilis* (8 corticosterone, 15 cortisol), 35 measurements from *R. imitator* (16 corticosterone, 19 cortisol), and 19 measurements from *R. variabilis* (3 corticosterone, 16 cortisol). While we had few measurements of corticosterone in *R. variabilis* samples, we include them in the analysis as valuable preliminary information about a species that, to our knowledge, has no current published glucocorticoid data.

**Table 1:** Results from linear model for hormone release rate across injection type and time points

	log(release rate, pg/h)		
*Predictors*	*Estimates*	*CI*	*p*
Intercept	4.93	3.54–6.32	**<0.001**
Snout-vent length (cm)	0.09	−0.22–0.39	**0.581**
Glucocorticoid type (cortisol)	−0.44	−0.87 to −0.01	**0.044**
Injection type (ACTH)	0.40	−0.09–0.88	0.105
Time point (post)	−0.59	−1.02 to −0.17	**0.007**
GC type x ACTH	0.06	−0.54–0.66	0.834
GC type x post-injection	0.62	0.01–1.23	**0.047**
ACTH x post-injection	0.96	0.38–1.55	**0.001**
GC type x ACTH x post-injection	−0.98	−1.81 to −0.14	**0.023**
**Random effects**			
σ^2^	0.29		
τ_00 trial:frogID_	0.12		
τ_00 frogID_	0.02		
ICC	0.33		
*N*_trial_	35		
*N*_frogID_	18		
Observations	109		
Marginal *R*^2^/Conditional *R*^2^	0.314/0.541		

*Note:* The reference groups are corticosterone, control (saline) injection, and pre-injection time point. *P* values <0.05 are listed in bold.

Finally, we compared the relationship between cortisol and corticosterone across species. Due to our limited sample sizes for the smaller *Ranitomeya*, we had too few samples with data for both cortisol and corticosterone release rate, so we restricted the comparison to the larger species (*D. tinctorius. P. terribilis*, and *E. anthonyi*). To compare with the *D. tinctorius* model (above), we fit separate linear models for *P. terribilis* (*n* = 7) and *E. anthonyi* (*n* = 12) with cortisol release rate as the response variable and corticosterone as a fixed effect.

## Results

### Glucocorticoid abundance and correlations in *D. tinctorius*

In *D. tinctorius*, corticosterone was statistically more abundant than cortisol in both plasma (ß = −2.04, *P* < 0.001; Fig. 1B) and water samples (ß = −0.55, *P* < 0.001; Fig. 1C). In plasma samples, corticosterone concentration was 22.8% higher than cortisol on average. In water samples, the difference between GC types was smaller; corticosterone release rate was 11.4% higher than cortisol on average.

When we compared water with plasma samples in *D. tinctorius*, the concentration of cortisol in plasma was positively correlated with the release rate of cortisol in water (ß = 1.68, *P* = 0.037; Fig. 1D). We found a slightly positive but statistically non-significant relationship between corticosterone concentration in plasma and corticosterone release rate in water (ß = 0.27, *P* = 0.39; Fig. 1D).

**Fig. 2:**
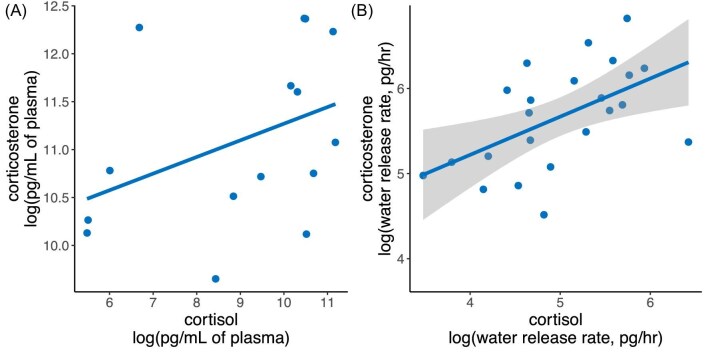
Correlation between glucocorticoids. Corticosterone and cortisol were positively correlated in both (A) plasma and (B) water. However, the relationship was only statistically significant in (B) water samples. Gray shaded area is the confidence interval around the statistically significant linear regression for water samples.

In both plasma and water, cortisol and corticosterone were positively correlated, but this relationship was weaker in plasma samples and only statistically significant in water samples (plasma: ß = 0.17, *P* = 0.15; water: ß = 0.45, *P* = 0.0077; Fig. 2).

### ACTH challenges

**Fig. 3:**
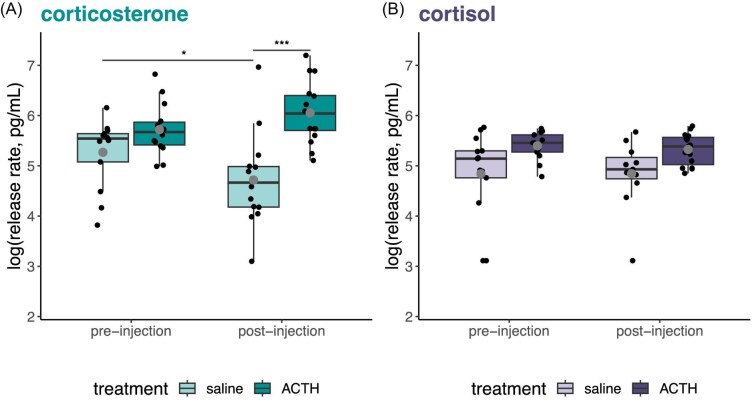
Glucocorticoid response to an ACTH injection compared to a saline control injection. (A) Corticosterone release rate was higher after an ACTH injection than after a saline injection. (B) In contrast, we found no difference in cortisol release rate between samples taken before and after injection of either saline or ACTH. Lighter colors (on the left within injection groups) indicate the control/saline injection for the respective glucocorticoid. Larger dots indicate the mean for each treatment group. Smaller dots are individual water samples. (* = *P* < 0.05, *** = *P* < 0.001).

The effect of an injection (ACTH vs. vehicle) varied by GC and injection type (GC × injection type × time point: ß = −0.97, *P* = 0.024; Table 1; Fig. 3). Frogs had higher corticosterone release rates after an injection of ACTH compared to an injection with saline (pairwise comparison of corticosterone between ACTH and saline post-injection: ß = 1.36, *z* = 5.86, *P* < 0.001; Fig. 3). This difference was driven primarily by a decrease in corticosterone in saline injected frogs (pairwise comparison of corticosterone between pre- and post-injection saline: ß = 0.59, *z* = 0.21, *P* = 0.031; Fig. 3) compared to a maintenance of corticosterone levels in ACTH injected frogs (ß = −0.36, *z* = −1.79, *P* = 0.34). Indeed, this difference between treatments also drove a main effect of decreased GC release rates post-injection (ß = −0.59, *P* = 0.007; Table 1). Cortisol release did not vary pre- and post-injection for either type of injection (pairwise comparisons of cortisol between pre- and post-injection: saline ß = −0.02, *z* = −0.09, *P* = 1.00, ACTH ß = −0.01, *z* = −0.03, *P* = 1.00; Fig. 3).

### Species variation in relative glucocorticoid abundance

**Fig. 4:**
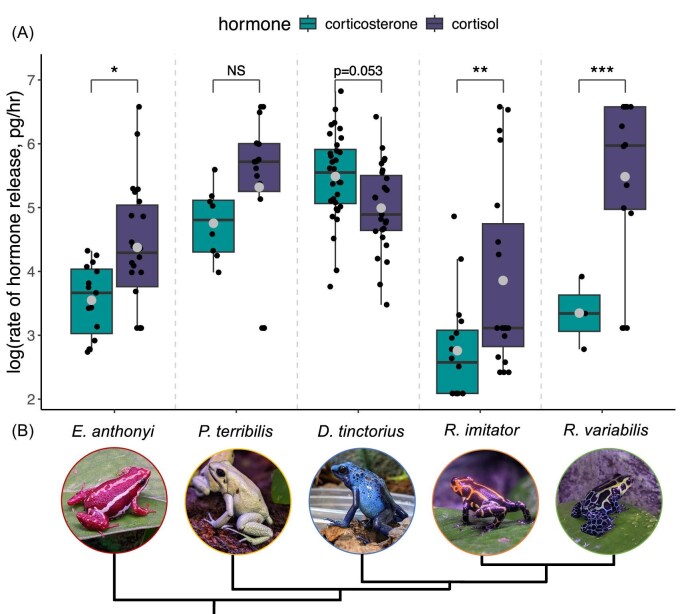
Species variation in relative glucocorticoid abundance. (A) For four out of the five species tested, the release rate of corticosterone was lower than the release rate of cortisol. In *D. tinctorius*, corticosterone was released 1.64 times more than cortisol. Larger dots indicate the mean for each group. Smaller dots represent individual samples (NS = not significant, * = *P* < 0.05, ** = *P* < 0.001, *** = *P* < 0.0001). (B) The species names correspond to the respective photos beneath the *x*-axis labels. The tree below the photos indicates the phylogenetic relationships between species (Guillory et al. 2019).

We found species differed in their release rate of each glucocorticoid (*F*_4,71.8_ = 9.57, *P* < 0.0001; Fig. 4). Specifically, pairwise comparisons within species revealed *D. tinctorius* released slightly more corticosterone (ß = −0.55, *P* = 0.053), while three species (*E. anthonyi. R. imitator*, and *R. variabilis*) released more cortisol (Table 2; Fig. 4). In *P. terribilis*, there was a trend for higher cortisol release than corticosterone, but the difference was not statistically significant (Table 2; Fig. 4). In a less conservative analyses, excluding all samples that did not fall within the range of the assays (i.e., remove censored values), the overall patterns were the same; however, the difference between GC types became statistically significant for *P. terribilis* (ß = 0.98, *P* = 0.020), which released 2.5× more cortisol than corticosterone.

**Table 2:** Results from simultaneous multiple pairwise comparisons of means for hormone release rate across species

	log(release rate, pg/h): cortisol—corticosterone				
*Species*	*Estimates*	*Std. error*	*z-value*	*P-value*	Cortisol is X times higher than corticosterone on average
*D. tinctorius*	−0.55	0.22	−2.55	**0.053**	**0.61**
*E. anthonyi*	**0.80**	**0.28**	**2.84**	**0.022**	**3.29**
*P. terribilis*	0.67	0.37	1.80	**0.31**	**2.4**
*R. imitator*	**1.08**	**0.29**	**3.67**	**<0.001**	**6.2**
*R. variabilis*	**2.29**	**0.57**	**4.02**	**<0.0001**	12.7*

*Note: P*-values <0.05 are in bold. *The difference for *R. variabilis* should be interpreted with caution due to small sample sizes.

**Fig. 5:**
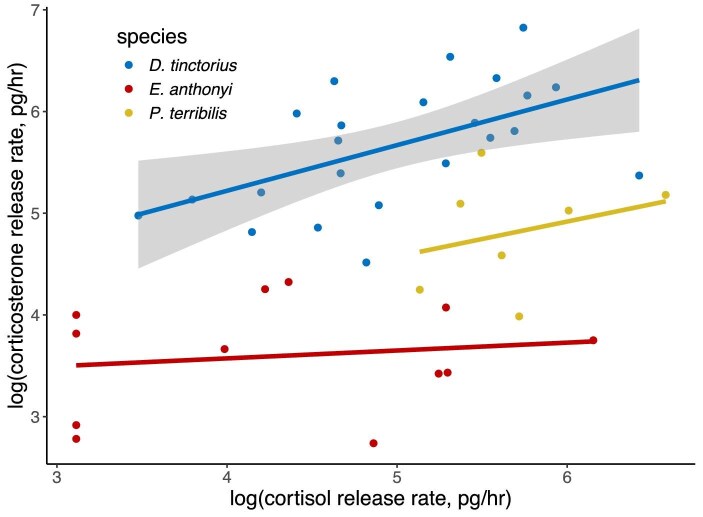
Species variation in the relationship between GC types. The positive relationship between cortisol and corticosterone was stronger in water samples. Gray shaded area is the confidence interval around the statistically significant linear regression for *D. tinctorius* water samples. *Dendrobates tinctorius* data are the same as those in Fig. 2. We added them here as well for ease of comparison across the species.

Unlike the positive correlation between cortisol and corticosterone in water samples from *D. tinctorius* (see Section “Glucocorticoid abundance and correlations in *D. tinctorius*”), the two GCs were not statistically significantly correlated in *P. terribilis* (ß = 0.24, *P* = 0.53; Fig. 5) and *E. anthonyi* (ß = 0.29, *P* = 0.65; Fig. 5).

## Discussion

Glucocorticoids are involved in many physiological, developmental, and behavioral processes that are essential for survival and fitness (Nicolaides et al. 2015; MacDougall-Shackleton et al. 2019); therefore, understanding glucocorticoids and HPA axis dynamics can inform research and conservation efforts and improve our understanding of how animals are impacted by and respond to environmental changes and threats (Millspaugh and Washburn 2004; Busch and Hayward 2009). Here, we present our findings on GC variation in poison frogs as an example of how measuring both cortisol and corticosterone can bring to light new information and spark more scientific inquiry.

Our initial goal was to identify which glucocorticoid was most abundant and responsive to activation of adrenocortical tissue via injection of ACTH, that is, the dominant GC by the classic definition, in our primary focal species, *D. tinctorius*. Our interest in the question was spurred generally by an increasing awareness of the subtleties of hormonal effects both within and across species, and specifically by our previous findings that increased cortisol is associated with parental care in adult *D. tinctorius* (Fischer and O’Connell 2020), yet corticosterone is more abundant and ACTH-responsive in *D. tinctorius* tadpoles (Surber-Cunningham et al. 2025). Given both GCs were measurable in *D. tinctorius*, we tested and confirmed non-canonical patterns in adults of four additional Dendrobatid species. Our findings challenge the common assumption that corticosterone is always more abundant in frogs and suggest that focusing only on the ACTH-responsive GC can overlook potentially important ACTH-independent regulation of other physiologically important GCs. Below, we discuss the biological implications, remaining technical challenges and limitations, and prospects of this work.

### Glucocorticoid abundance and dominance in *D. tinctorius*

We used non-invasive waterborne hormone sampling to quantify cortisol and corticosterone excretion rates in adult *D. tinctorius*. In many other species, including *D. tinctorius* tadpoles (Surber-Cunningham et al. 2025), one GC is substantially more abundant. In contrast, in adult *D. tinctorius* water samples, corticosterone was almost equally as abundant as cortisol. As a substantially greater abundance often predicts ACTH responsivity, this unexpectedly small difference between cortisol and corticosterone and previous evidence for an association between cortisol and behavior in adult *D. tinctorius* (Fischer and O’Connell 2020) left open the question of GC dominance by the typical definition of ACTH responsivity.

In response to an ACTH challenge, we found evidence for corticosterone as the “dominant” GC in *D. tinctorius*. In a within subjects, counter-balanced design, the release rate of corticosterone was higher following an ACTH injection than a saline injection, regardless of whether the saline injection was the first or second injection the frogs experienced. Interestingly, this ACTH vs. saline group difference resulted primarily from a marked decrease in corticosterone excretion in the saline group. This pattern suggests that all frogs mount a stress response to sampling but, while this response dissipates in the control group, it is sustained following ACTH injection (Fig. 3). Interestingly, cortisol did not respond to either an injection of ACTH or saline (Fig. 3) suggesting cortisol is not responsive to acute adrenal activation and does not fluctuate across short time periods (i.e., does not show an increased or habituation to waterborne sample collection). In combination, these results indicate corticosterone is more responsive to acute adrenal activation than cortisol, despite similar amounts of cortisol and corticosterone prior to injections.

Often, the GC that responds most to adrenal activation through an ACTH challenge is also more abundant (e.g., Forsburg et al. 2019; Hopkins et al. 2020). While this pattern holds in *D. tinctorius*, the difference in abundance between the GCs was relatively small compared to other species. For example, across 18 species of mammals cortisol is more responsive to ACTH and cortisol concentrations were between 7.5 and 49 times higher than corticosterone (Koren et al. 2012). Similarly, in the American bullfrog (*R. catesbeiana*), corticosterone is ACTH responsive (Krug et al. 1983) and ∼26 times as abundant than cortisol (Krug et al. 1983; Wright et al. 2003). Our finding that corticosterone and cortisol are similar in abundance yet differentially responsive to ACTH underscores the need for future studies that characterize both GCs to better understand their role in poison frog physiology and behavior.

Evidence for corticosterone as the ACTH-responsive GC in *D. tinctorius* aligns with our findings in *D. tinctorius* tadpoles (Surber-Cunningham et al. 2025), a previous study using restraint stress in the mimic poison frog (*R. imitator*; Nowicki et al. 2024), and broader taxonomic trends in amphibians. However, our previous findings also suggest a functional role for cortisol in parental care (Fischer and O’Connell 2020). We hypothesize that corticosterone and cortisol may serve distinct functions and may be released via distinct mechanisms depending on context, thus providing a means for modular regulation of behavioral and physiological functions of hormones (McGlothlin and Ketterson 2008). Though speculative, this hypothesis aligns with evidence from other taxa for distinct roles of the two GCs in different tissues (Schmidt and Soma 2008), differential binding affinities of the two GCs with their shared receptors (Sutanto and De Kloet 1987; Katsu et al. 2016), and differential responsiveness of the two GCs to acute vs. chronic stressors (Venkataseshu and Estergreen 1970; Vera et al. 2011, 2012; Koren et al. 2012). There are various non-ACTH routes of GC production, including regulation via neurotransmitters and neuropeptides involved in behavior (Bornstein and Chrousos 1999), which provide a potential mechanism for modularity in GC release. Further, while both GCs bind to the same receptor types, the potential for differential release mechanisms poses an intriguing reframing of our understanding of how the HPA axis functions. For example, the parental state involves a myriad of physiological changes (Rosenblatt and Snowdon 1996; Numan 2020) and perhaps the long-term physiological versus short-term behavioral demands of parenting rely on the production and localization of glucocorticoids through different routes. We suggest future studies explore the idea that ACTH and ACTH-independent mechanisms could provide modular regulation of responses to various stressors and stressful contexts, such as parental care.

Though our interpretations remain speculative, there is growing precedent for the differential functionality of cortisol and corticosterone within species (Schmidt and Soma 2008; Vera et al. 2017). In some species and contexts, cortisol is more responsive to acute stress than corticosterone (Venkataseshu and Estergreen 1970; Koren et al. 2012; Vera et al. 2012, 2011); however, the two GCs may also simultaneously elevate in more chronic stress states with high energetic burdens. For instance, both cortisol and corticosterone increase during pregnancy in humans (Wintour et al. 1978), little brown bats (*Myotis lucifugus*, Reeder et al. 2004), North Atlantic right whales (Hunt et al. 2017), and Asian elephants (*Elephas maximus*, Kajaysri and Nokkaew 2014). In essence, the ratio of the two GCs in circulation may be dependent on physiological and/or behavioral state. In the context of increased cortisol during parenting in *D. tinctorius* (Fischer and O’Connell 2020), an increase in corticosterone, but not cortisol, following an ACTH injection suggests non-ACTH routes of GC production may be important in upregulating cortisol during behavioral states with higher energetic demand, such as tadpole transport. Such modular regulation could occur through changes in the regulation of the enzyme required to produce cortisol (17α-hydroxylase). Further studies are needed to explore these intriguing possibilities.

The motivation to measure both glucocorticoids also stems from our observations and the results of others (e.g., Koren et al. 2012) demonstrating that the two GCs are not always correlated with one another nor entirely redundant in function. In non-invasive water samples from *D. tinctorius*, cortisol was positively correlated with corticosterone. However, this correlation was not significant in plasma samples or in water samples from *P. terribilis* or *E. anthonyi*. These results suggesting the ratio of circulating GCs may fluctuate and the relationship between the GCs may vary by sampling matrix, for example, due to differences in excretion rates. Another possibility yet to be explored is temporal variation in the regulation of each GC. If the abundance of one GC is more dynamic over short time periods than the other, this could lead to a more stable positive relationship between the GCs in cumulative samples, such as water, than the more immediate sampling of plasma. Though more work is needed in *D. tinctorius*, there is growing precedent for the differential functionality of cortisol and corticosterone within species (Schmidt and Soma 2008; Vera et al. 2017), and our findings suggest several avenues for future research exploring the practical and functional consequences of patterns we report here.

### Species variation in glucocorticoids

Following our unexpected results in *D. tinctorius*, we characterized waterborne hormone levels in four additional poison frog species, yielding data from five species representing four genera. Results from all five species across the *Dendrobatidae* family contradict the common assumption that corticosterone is the more abundant GC in amphibians generally (Moore and Jessop 2003; Wingfield and Romero 2011; Norris and Carr 2013) and in terrestrial amphibians specifically (Jungreis et al. 1970). In waterborne hormone samples, we found cortisol was released at a higher rate than corticosterone in all four additional species, though the magnitude of this difference varied (2.4–12.7×; Fig. 4). Notably, Baugh et al. (2018) found similar results using HPLC-MS methods to measure GCs in water samples from the terrestrial túngara frog. We opportunistically chose these five species and systematic sampling is needed to determine whether variance in relative GC abundance across Dendrobatids is associated with phylogeny, life history, or some combination. Nonetheless, we can confidently say that this group does not follow the general assumption of higher corticosterone abundance in (terrestrial) amphibians.

Most studies comparing cortisol and corticosterone within-species have been conducted in mammals, with few systematic studies in other taxa (Vera et al. 2017). The limited number of studies in amphibians that have looked at both cortisol and corticosterone report a range of empirical results across different sample types with no consistent pattern (see above and review in the introduction). These findings in amphibians and other taxa beg the question of what explains this variation in GC abundance. One idea is that the two GCs are functionally interchangeable, and variation is therefore random. However, given variation even within species, it has been suggested that cortisol and corticosterone may in fact have some different physiological functions and be differentially regulated (Koren et al. 2012; Vera et al. 2019), as suggested by our observations in *D. tinctorius*. The two GCs could be playing different roles across age classes, as suggested by our findings that corticosterone is seven times as abundant as cortisol in tadpoles (Surber-Cunningham et al. 2025), but the two GCs are only slightly different in adults of *D. tinctorius*. Further exploration of age-related changes is particularly interesting in amphibians that have a unique biphasic life cycle in which corticosterone is known to play an essential role in metamorphosis (Krug et al. 1983; Jaudet and Hatey 1984; Kulkarni and Buchholz 2014; Shewade et al. 2020). A non-mutually exclusive alternative is that the two GCs could have distinct metabolic functions. For instance, in a study on zebra finches (*Taeniopygia guttata*), the more abundant GC varied by tissue type with more corticosterone (the widely-assumed dominant GC of birds) detected in plasma, but more cortisol detected in immune tissues (Schmidt and Soma 2008). Additionally, corticosterone and cortisol may differentially bind to species-specific corticosteroid-binding globulins resulting in differing amounts of bound and unbound corticosteroid available in circulation (Martin and Ozon 1975; Westphal 1986; Breuner and Orchinik 2002). Pharmacological studies also suggest differential glucocorticoid receptor-mediated gene regulation based on which type of corticosteroid is available (e.g., Pariante et al. 2003; Nixon et al. 2016) and differential transport of each corticosteroid across the blood-brain barrier (e.g., Karssen et al. 2001). In sum, focusing only on the ACTH-responsive or “dominant” GC ignores ACTH-independent regulation or extra-adrenal production of the other GC, which may be just as physiologically important (Bornstein and Chrousos 1999; Bornstein et al. 2008; Taves et al. 2011).

### Caveats, considerations, and future directions

We end by discussing several caveats and considerations about our case study that provide interesting areas for future study. First, we note that many assumptions about GC abundance and dominance are based primarily on studies of plasma or adrenocortical tissue. It is possible that these assumptions do not translate to waterborne hormone sampling. For example, cortisol and corticosterone release rates could differ across species such that waterborne levels of GCs are not representative of circulating levels of GCs in all species. We think it is unlikely that mechanism leading to a difference in release rates would be different among closely related species, thus the differences we see among species likely reflect actual physiological differences. Additionally, there is precedent for our findings as previous studies examining ACTH responsiveness and relative abundance of GCs across many different sample types in amphibians—including water, fecal, saliva, and skin swabs—have demonstrated variation in GC dominance across species (e.g., Baugh et al. 2018; Hammond et al. 2018; Narayan et al. 2019; Hopkins et al. 2020). In any case, our results provide evidence that the Dendrobatid species we tested produce substantial amounts of cortisol, contrary to general assumptions that amphibians only produce corticosterone. Additional work will clarify the mechanistic origins and potential functional consequences of differences in relative GC abundance across timescales, stressor types, and species.

Second, waterborne hormones are an integrative measure of hormone release rate over time, rather than a measurement of a moment in time as in plasma. Thus, the collection of waterborne hormones and plasma is inherently separated in time and therefore, perhaps our null expectation should not be a tight relationship between plasma and waterborne hormones. For instance, the duration of waterborne hormone collection may impact the strength of the correlation between plasma and water (e.g., shorter collection timeframes may be more correlated with plasma).

Alternatively, even when plasma and water hormone levels are correlated, GCs may differ in their release rates such that relative abundances differ between plasma and water. For example, in túngara frogs (*Physalaemus pustulosus*), cortisol was present in higher concentrations than corticosterone in waterborne samples, but corticosterone was higher in plasma samples (Baugh et al. 2018). Alternative non-lethal methods for measuring hormones, such as collecting saliva (Hammond et al. 2018, 2020; Tornabene et al. 2023), may be better correlated with plasma hormones given both techniques give a snapshot of hormone concentrations, yet this remains to be tested. In any case, even when correlations between waterborne and plasma samples are imperfect, waterborne samples provide a non-invasive method for measuring biologically meaningful differences in glucocorticoids in anurans (reviewed in Ruthsatz et al. 2023) and understanding the complexities of this relationship provides interesting opportunities for further study.

Finally, there are inherent limitations with non-lethal hormone sampling. For example, in our experience using 1-h sampling, hormones are generally less abundant in water samples than plasma samples and thus can be more difficult to measure. Limited abundance of hormones also makes re-measurement of samples challenging as the concentration can only be diluted so much while remaining within detectable limits of the assay. We had several water samples that did not measure in the range of our ELISA standards. Most of these samples were for our smallest *Ranitomeya* species and we chose to conservatively include them as censored values. While the exact concentration of hormone was not known, the fact that we could confidently determine whether the value was too high or too low to quantify by our curve still provided valuable information. We note that corticosterone, but not cortisol, was particularly difficult to measure in samples from *R. variabilis*, likely due to much lower concentrations of excreted corticosterone than cortisol. Excluding the censored values reduced our sample sizes but did not change the overall patterns we observed, suggesting there is a robust trend for higher cortisol than corticosterone in multiple species of poison frogs. Despite the limitations of non-invasive methods, these methods are critical in species of conservation concern and small-bodied species where non-lethal techniques are necessary for studies on GC physiology.

### Conclusions

Our findings suggest interesting, under-appreciated, and unexplored variation in HPA activity and glucocorticoids across Dendrobatid poison frogs. These findings highlight the value of measuring both cortisol and corticosterone when investigating the complex, multivariate roles of GCs in physiology, development, and behavior, inspiring more questions than they answer. Even if only one GC increases in response to a stressor, the other GC can still play an important role, and we encourage more researchers to investigate both GCs in their focal species. While measuring additional steroids adds cost and can be complicated by measurement of low abundance samples (such as our own), measuring both GCs in preliminary and/or follow-up studies can be valuable. Even in well-studies species, measuring both GCs can bring to light new information about the differential regulation and functionality of cortisol and corticosterone, which may not be as interchangeable as we assume (Hancock 2010). With data across more species, we can compare patterns of GC dominance and abundance to investigate adaptive and/or ecological explanations for variation across species, populations, and life stages, and test hypotheses about potential unique physiological roles for cortisol vs. corticosterone. Additionally, measuring both cortisol and corticosterone may better inform conservation practices through improving our knowledge about stress physiology across species and environments. By understanding the physiological stress processes, we can better consider how to improve conservation efforts to minimize potential stress-related fitness consequences which could impact the species’ survival (Hofer and East 1998; Busch and Hayward 2009).

## Data Availability

The data underlying this article are available on FigShare, at https://doi.org/10.6084/m9.figshare.23537409.v2.
